# Supplementary figures and images for: The plasmid-borne hipBA operon of Klebsiella michiganensis encodes a potent plasmid stabilization system
Source: J Appl Microbiol. 2024 Sep 20;135(10):lxae246. doi: 10.1093/jambio/lxae246 (PMC11487325; doi:10.1093/jambio/lxae246)

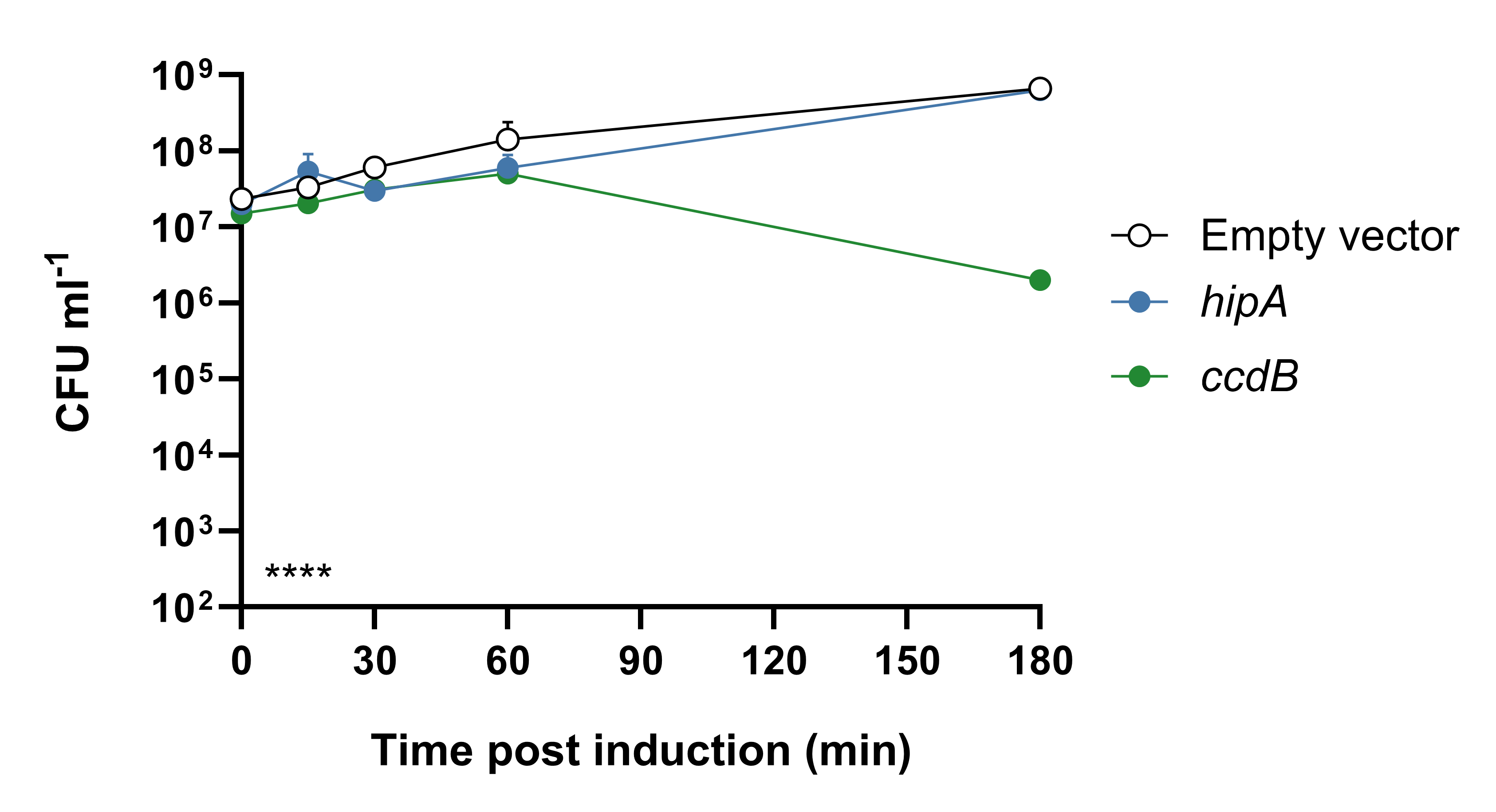

Supplement: lxae246_Supplemental_Files [file lxae246_supplemental_files.zip › Supplementary Figure 1.tif]

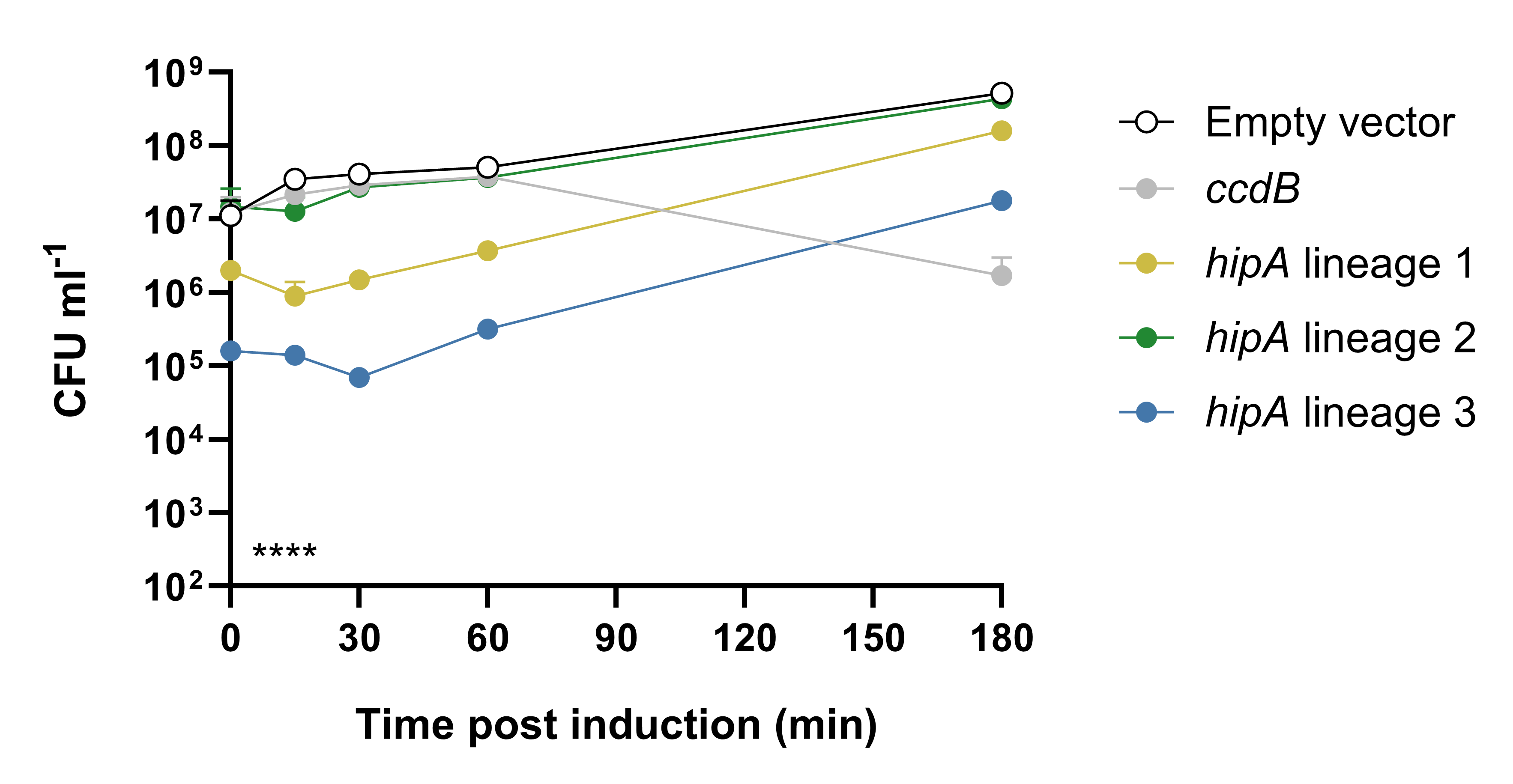

Supplement: lxae246_Supplemental_Files [file lxae246_supplemental_files.zip › Supplementary Figure 2.tif]

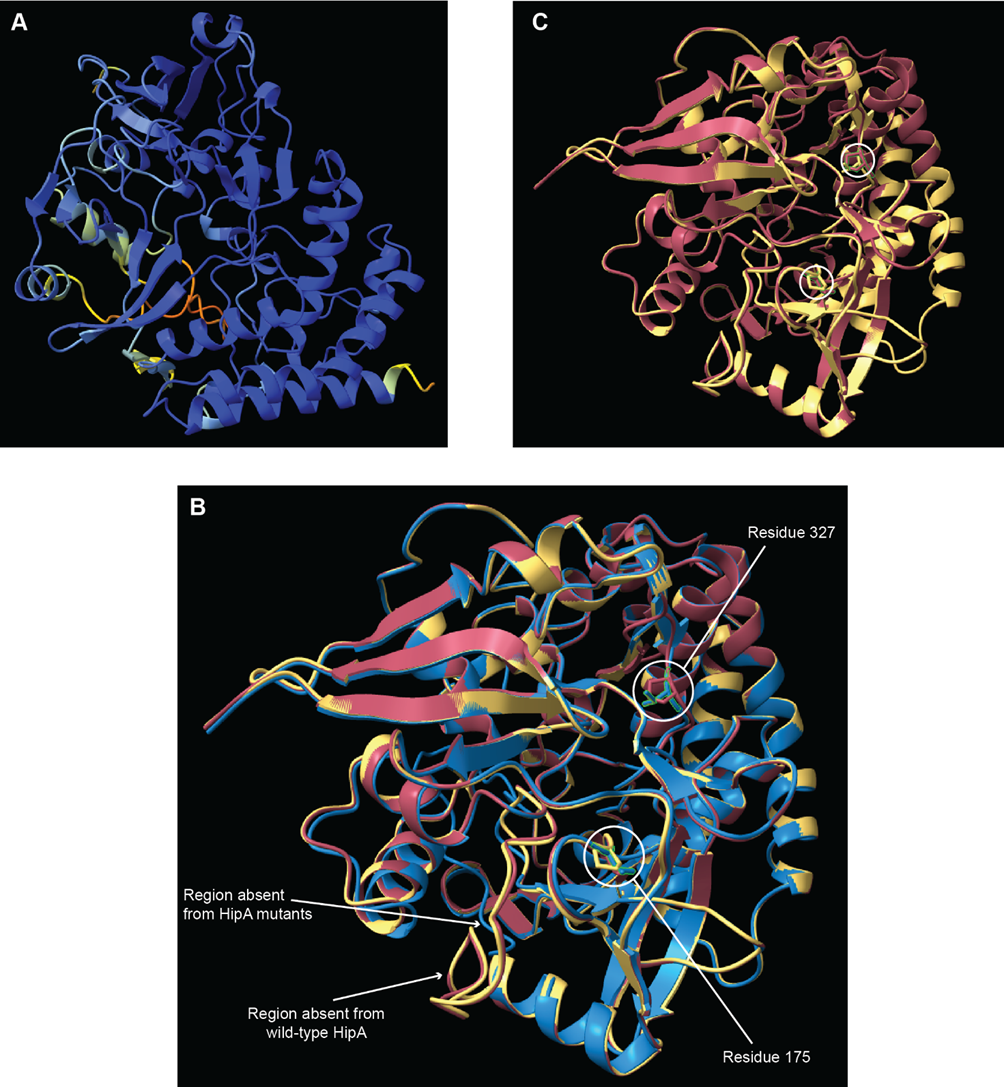

Supplement: lxae246_Supplemental_Files [file lxae246_supplemental_files.zip › Supplementary Figure 3.tif]

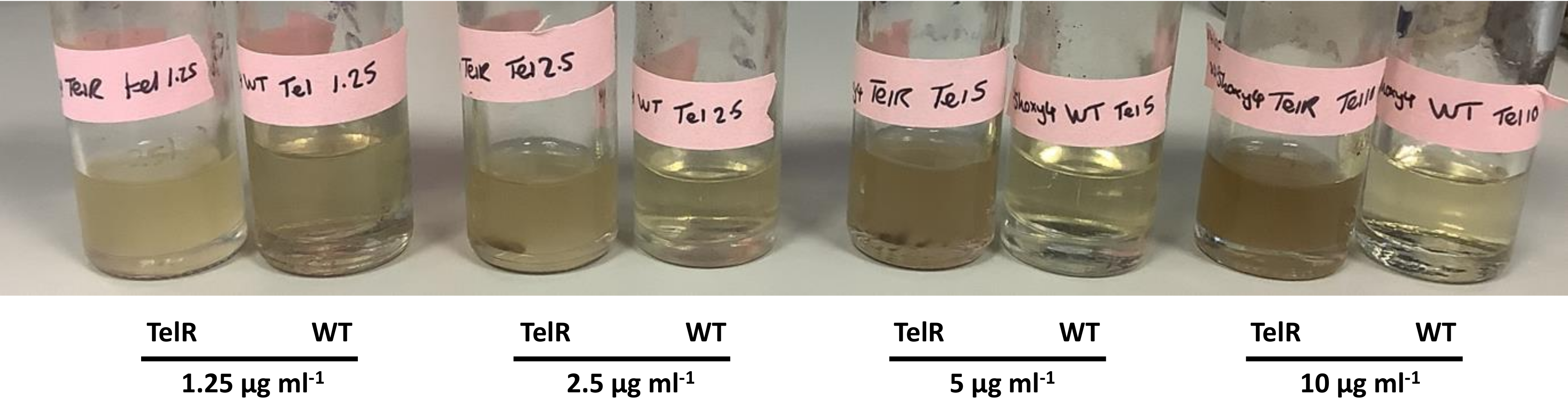

Supplement: lxae246_Supplemental_Files [file lxae246_supplemental_files.zip › Supplementary Figure 4.tif]

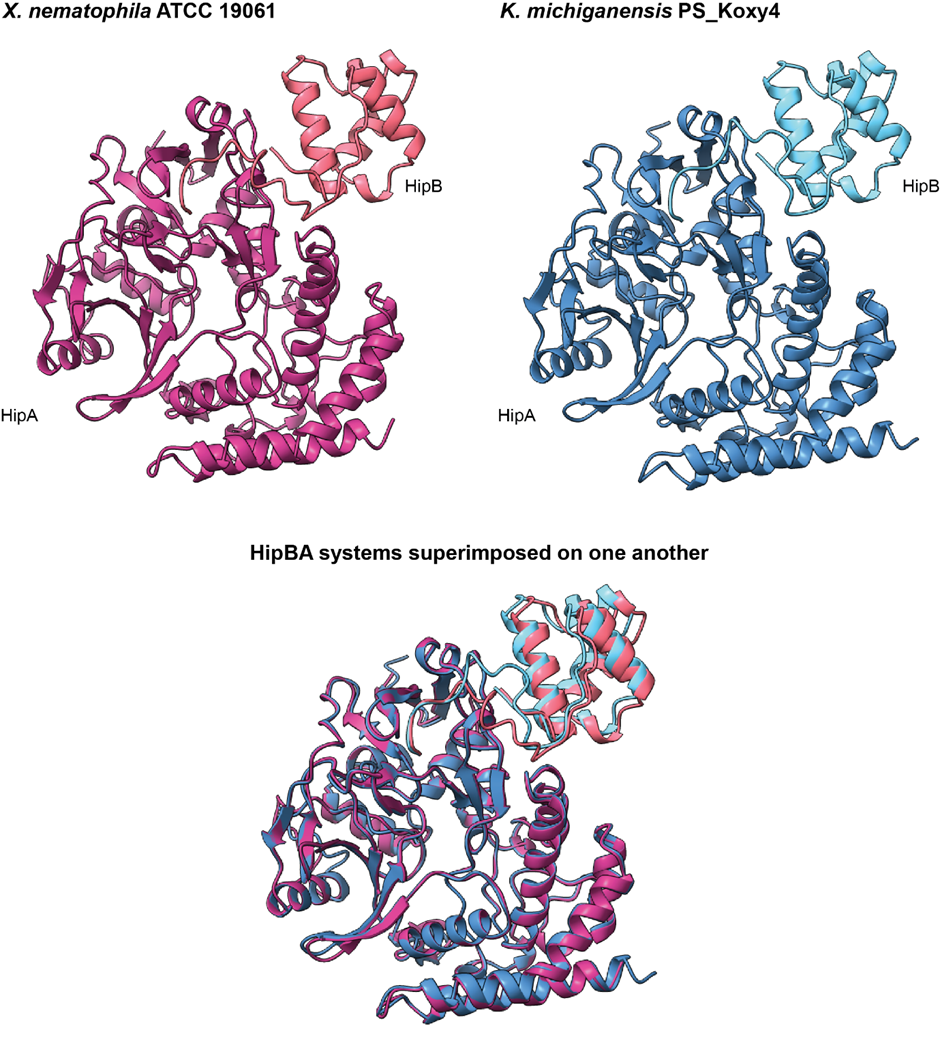

Supplement: lxae246_Supplemental_Files [file lxae246_supplemental_files.zip › Supplementary Figure 5.tif]
